# Detecting neurodevelopmental trajectories in congenital heart diseases with a machine-learning approach

**DOI:** 10.1038/s41598-021-82328-8

**Published:** 2021-01-28

**Authors:** Elisa Cainelli, Patrizia S. Bisiacchi, Paola Cogo, Massimo Padalino, Manuela Simonato, Michela Vergine, Corrado Lanera, Luca Vedovelli

**Affiliations:** 1 Department of General Psychology, University of Padova, Padua, Italy; 2 Padova Neuroscience Centre, PNC, Padua, Italy; 3 Department of Medicine, Clinica Pediatrica, University Hospital S Maria Della Misericordia, University of Udine, Udine, Italy; 4 Pediatric and Congenital Cardiovascular Surgery Unit, Department of Cardiac, Thoracic and Vascular Sciences, Padova University Hospital, Padua, Italy; 5 PCare Laboratory, Fondazione Istituto Di Ricerca Pediatrica “Citta Della Speranza”, Padua, Italy; 6 Unit of Biostatistics, Epidemiology, and Public Health, Department of Cardiac, Thoracic, Vascular Sciences, and Public Health, University of Padova, Padua, Italy

**Keywords:** Neuroscience, Psychology, Cardiology

## Abstract

We aimed to delineate the neuropsychological and psychopathological profiles of children with congenital heart disease (CHD) and look for associations with clinical parameters. We conducted a prospective observational study in children with CHD who underwent cardiac surgery within five years of age. At least 18 months after cardiac surgery, we performed an extensive neuropsychological (intelligence, language, attention, executive function, memory, social skills) and psychopathological assessment, implementing a machine-learning approach for clustering and influencing variable classification. We examined 74 children (37 with CHD and 37 age-matched controls). Group comparisons have shown differences in many domains: intelligence, language, executive skills, and memory. From CHD questionnaires, we identified two clinical subtypes of psychopathological profiles: a small subgroup with high symptoms of psychopathology and a wider subgroup of patients with ADHD-like profiles. No associations with the considered clinical parameters were found. CHD patients are prone to high interindividual variability in neuropsychological and psychological outcomes, depending on many factors that are difficult to control and study. Unfortunately, these dysfunctions are under-recognized by clinicians. Given that brain maturation continues through childhood, providing a significant window for recovery, there is a need for a lifespan approach to optimize the outcome trajectory for patients with CHD.

## Introduction

Congenital heart diseases (CHDs) are the most common congenital defects, affecting nearly 1% of all newborns^1^. Advances in prenatal diagnosis, perioperative management, and postoperative care have dramatically increased the population of CHD survivors. However, CHD patients are at risk of long-term developmental impairments. Indeed, the high survival rate of these children into adulthood has determined an alarming prevalence of long-term sequelae^2^.

Recent investigations have shown that even patients with average intelligence and without neuromotor impairments may exhibit subtle isolated neuropsychological difficulties and psychological disorders^3,4^. These impairments and dysfunctions impact one’s social life and academic performance negatively, with long-lasting consequences on career and socioeconomic well-being in adulthood^5^. However, in clinical practice, these problems are often underestimated or not promptly recognized.

The underlying pathophysiological mechanism of CHDs is complex and not yet fully understood. It has been demonstrated that CHD may interfere with brain development, as reported by several imaging studies showing widespread brain abnormalities in CHD term neonates^6^. The cardiovascular and nervous systems are interconnected and regulate each other during development. Not surprisingly, these systems are functionally interconnected in both health and disease.

This already-complex condition is also influenced by many other variables, such as patient-specific characteristics, potential hypoxic/ischemic cascades triggered by hypoperfusion during cardiac surgery, and pre- and postoperative factors^7,8^. Furthermore, the effects of early exposure to stressful environments and dysfunctional parental attitudes associated with a child’s medical illness may be additional risk factors, arguments thoroughly discussed in other medical contexts (for example, see^9^).

Unfortunately, we lack precise prognostic tools in terms of CHD; some biomarkers appear promising^7,10,11^, and CHD severity has been generally associated with an increased incidence of impairments, but studies do not completely agree^2,12^. The impact of medical factors on developmental trajectories have not been well explored yet, and only a few data on specific CHD populations are available^3,13^.

In this study, we performed a neuropsychological and psychopathological assessment of children with CHD, comparing their performances with those of age-matched control children. We implemented a machine-learning approach to identify and rank the most important parameters that differentiated the two groups. To identify disease subtypes, we also performed a cluster analysis to divide individual CHD patients into homogenous subgroups based on psychological profiles. Finally, we looked for an association of psychological outcomes with several medical parameters.

Patients’ selection was unrelated to CHD type; selection was only based on the patients’ age at recruitment to increase the generalizability of results across heart defects (Table 1).

**Table 1:** Clinical characteristics of the CHD population.

Clinical characteristics	N = 37
**STAT, n (%)**	
1	5 (13%)
2	15 (41%)
3	7 (19%)
4	7 (19%)
5	3 (8%)
Age at surgery (months)	15.2 ± 20.3 (0–66)
Pre-surgery oxygen saturation (%)	90.44 ± 7.41 (74.0–100)
Temperature nadir (°C)	30.2 ± 3.9 (18.0–35.3)
CPB time (min)	127 ± 49.6 (50–250)
CPB time with cerebral saturation < 45% (%)	19.4 ± 27.8 (0–100)
Arterial plasma lactates (mmol/L)	2.6 ± 1.5 (0.9–6.9)
Intensive care unit stay (days)	6.6 ± 10.7 (1–62)
Prematurity, n (%)	2 (5%)

Continuous variables are expressed as mean ± standard deviations (range min–max).

STAT: The Society of Thoracic Surgeons-European Association for Cardio-Thoracic Surgery score CPB: cardiopulmonary bypass.

## Results

### Descriptive results

At the time of the study, 75 children were eligible; 20/75 were lost at follow up or declined to participate, 6/75 were excluded because they did not speak Italian, and 7/75 because of medical comorbidities; finally, 3/42 did not attend all scheduled meetings and therefore did not complete evaluations, and 2/42 additional patients were excluded because of IQ (< 70). Of the 38 children not included in the study, 6 (15%) was STAT 1, 15 (39%) STAT 2, 8 (21%) STAT 3, 6 (15%) STAT 4, 3 (7%) STAT 5.

The final sample population consisted of 37 patients (median age 7.41, 5.53–7.91 years, males 21, 57%).

We recruited 34 healthy children (from here, called “controls “) (median age 7.25, 6.00–8.25 years, males 12, 35%). From the comparison of neuropsychological scores, 3 CHD children and one control child were excluded because of missing data. Cluster analysis was performed on 34 CHD children for the same reason.

Five CHD patients obtained borderline IQ scores (70 ≥ IQ < 85); all other CHD children demonstrated IQs in the average range. All control children demonstrated IQs in the average range.

Details on cognitive and neuropsychological results in both groups are shown in Table 2.

**Table 2:** Median and mean cognitive and neuropsychological scores obtained by the control and CHD groups.

Domain	Test	Controls (N = 33)	CHD (N = 35)	Adjusted P value
**General intelligence**	**QI**			**0.041**
	Median (Q1,Q3)	105 (101, 116)	99 (89.5, 110)	
**Language**	**Naming**			**0.026**
	Median (Q1,Q3)	0.1 (0.0, 0.7)	− 0.3 (-0.7, 0.2)	
**Attention**	**Visual attention**			0.242
	Median (Q1,Q3)	11.0 (10.0, 12.0)	10.5 (8.0, 12.0)	
	**Auditory attention**			** < 0.001**
	1	0	4	
	2	2	6	
	3	2	13	
	4	22	5	
	5	4	7	
	6	3	0	
**Executive functions**	**Coding**			0.124
	Median (Q1,Q3)	10.0 (9.0, 13.0)	9.0 (7.0, 11.0)	
	**Digit span**			0.066
	Median (Q1,Q3)	10.0 (9.0, 11.0)	8.0 (7.0, 11.5)	
	**Semantic fluency**			** < 0.001**
	Median (Q1,Q3)	− 0.3 (-0.8, 0.6)	− 1.4 (− 2.1, − 0.7)	
**Memory**	**Visual memory**			**0.026**
	Median (Q1,Q3)	10.0 (10.0, 11.0)	9.0 (6.0, 10.0)	
**Social skills**	**Theory of mind A**			0.890
	Median (Q1,Q3)	0.1 (− 0.6, 1.0)	0.3 (− 0.5, 0.9)	
	**Theory of mind B**			0.242
	Median (Q1,Q3)	0.1 (− 0.3, 0.5)	− 0.4 (− 0.9, 0.8)	
	**Theory of mind T**			0.585
	Median (Q1,Q3)	10.0 (8.0, 12.0)	10.0 (8.0, 11.5)	
	**Affect recognition**			0.190
	Median (Q1,Q3)	10.0 (9.0, 11.0)	9.0 (4.5, 11.0)	

Details on psychopathological scores in all domains are shown in Tables 3 and 4.

**Table 3:** CBCL Median and mean scores in the control and CHD groups.

CBCL subscales	Controls (N = 34)	CHD (N = 37)	Adjusted P value
**Withdrawal**			**0.006**
Median (Q1,Q3)	50.0 (46.0, 52.0)	52.0 (50.0, 59.5)	
Mean	50.1 (8.6)	55.0 (6.6)	
**Somatic complaints**			**0.007**
Median (Q1,Q3)	50.0 (50.0, 51.0)	53.0 (50.0, 58.0)	
Mean	51.6 (9.2)	56.1 (7.5)	
**Anxiety/depression**			** < 0.001**
Median (Q1,Q3)	50.0 (47.0, 50.8)	52.0 (51.0, 60.5)	
Mean	50.1 (8.1)	55.2 (6.7)	
**Social problems**			0.076
Median (Q1,Q3)	51.0 (50.0, 56.0)	52.0 (51.0, 58.0)	
Mean	51.6 (8.3)	54.5 (5.1)	
**Thought problems**			**0.008**
Median (Q1,Q3)	47.0 (46.0, 51.0)	51.0 (50.0, 56.0)	
Mean	50.7 (6.1)	54.2 (6.3)	
**Attention problems**			** < 0.001**
Median (Q1,Q3)	50.0 (45.2, 51.0)	53.0 (50.0, 63.5)	
Mean	49.8 (7.0)	57.1 (7.8)	
**Rule breaking**			**0.049**
Median (Q1,Q3)	50.0 (43.2, 55.0)	52.0 (50.0, 56.0)	
Mean	50.3 (7.7)	53.7 (5.1)	
**Aggressive behavior**			**0.002**
Median (Q1,Q3)	50.0 (45.0, 50.8)	51.0 (50.0, 56.0)	
Mean	48.6 (6.5)	54.4 (8.1)	
**Internalizing**			**0.014**
Median (Q1,Q3)	44.0 (40.2, 49.8)	49.5 (45.2, 61.5)	
Mean	45.7 (10.5)	52.3 (12.1)	
**Externalizing**			**0.008**
Median (Q1,Q3)	43.0 (38.2, 50.0)	50.0 (44.0, 56.0)	
Mean	44.6 (8.3)	50.8 (10.8)

**Table 4:** CRS-R Median and mean scores in the two groups are reported.

CRS-R subscales	Controls (N = 34)	CHD (N = 37)	Adjusted P value
**Oppositional**			0.286
Median (Q1,Q3)	45.0 (40.0, 50.0)	47.5 (43.0, 55.0)	
Mean	46.1 (7.3)	51.0 (13.1)	
**Cognitive problems**			0.286
Median (Q1,Q3)	44.5 (41.2, 50.0)	48.5 (43.0, 54.8)	
Mean	48.1 (9.7)	52.1 (12.4)	
**Hyperactivity-impulsivity**			0.094
Median (Q1,Q3)	42.5 (40.0, 45.8)	49.5 (41.2, 56.0)	
Mean	44.0 (5.7)	51.3 (11.5)	
**Anxious-shy**			0.676
Median (Q1,Q3)	44.0 (39.0, 50.8)	47.0 (40.0, 52.5)	
Mean	46.3 (8.3)	48.1 (11.3)	
**Perfectionism**			0.290
Median (Q1,Q3)	41.5 (38.5, 49.0)	46.0 (40.0, 52.0)	
Mean	44.4 (7.9)	47.8 (11.3)	
**Social problems**			0.623
Median (Q1,Q3)	44.0 (43.0, 49.0)	44.0 (43.0, 47.8)	
Mean	47.3 (6.8)	47.2 (9.2)	
**Psychosomatic**			0.290
Median (Q1,Q3)	44.0 (42.2, 50.0)	44.0 (43.0, 57.2)	
Mean	46.3 (4.8)	52.1 (14.4)	
**ADHD Index**			0.269
Median (Q1,Q3)	44.5 (40.0, 56.0)	49.0 (42.2, 60.0)	
Mean	48.3 (10.1)	52.6 (12.5)	
**CGI: hyperactivity-imp**			0.136
Median (Q1,Q3)	44.5 (40.2, 49.8)	50.0 (42.0, 60.8)	
Mean	46.1 (8.3)	52.3 (12.5)	
**CGI: emotional instability**			0.442
Median (Q1,Q3)	42.0 (41.0, 47.0)	42.0 (41.0, 53.2)	
Mean	44.4 (6.0)	49.3 (13.4)	
**CGI: total**			0.190
Median (Q1,Q3)	43.5 (39.0, 47.0)	47.0 (41.0, 59.8)	
Mean	44.9 (7.7)	51.3 (13.7)	
**DSM IV: inattentive**			0.351
Median (Q1,Q3)	45.5 (42.0, 55.8)	47.0 (42.5, 57.2)	
Mean	48.6 (9.5)	52.1 (12.2)	
**DSM IV: hyperactivity-imp**			0.136
Median (Q1,Q3)	43.0 (41.0, 49.8)	49.0 (42.0, 60.5)	
Mean	44.5 (6.5)	51.2 (11.3)	
**DSM IV: total**			0.269
Median (Q1,Q3)	43.5 (41.0, 51.5)	48.5 (41.5, 61.5)	
Mean	46.4 (8.1)	51.5 (12.2)	

ADHD: attention deficit hyperactivity disorder; CGI: clinical global index; DSM: diagnostic and statistical manual of mental disorders.

Compared results of neuropsychological and psychopathological scores are reported in Tables 2, 3, and 4.

### Machine-learning features selection

**Figure 1:**
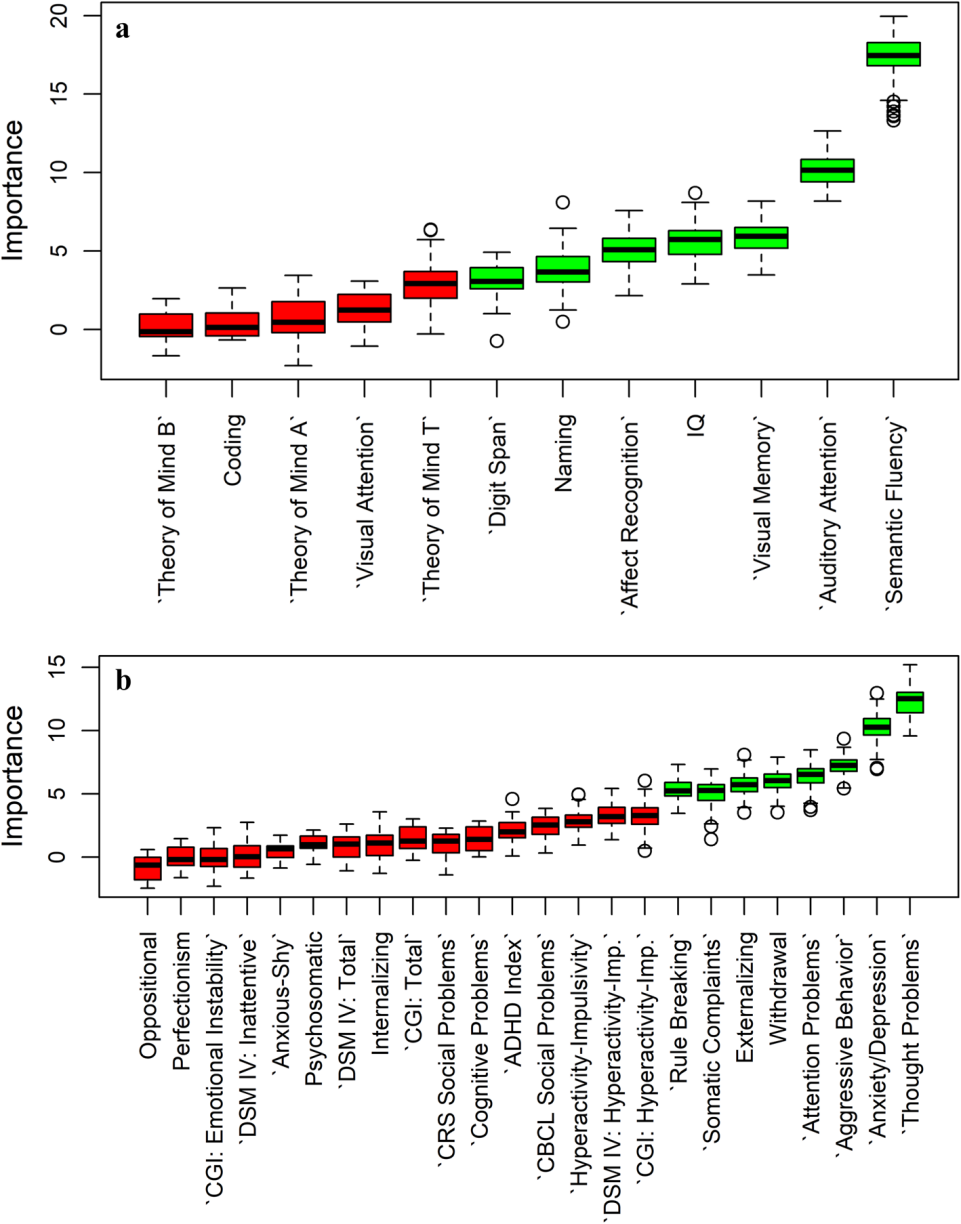
We applied the machine-learning algorithm (Boruta) to find the most influential features that distinguish between CHD patients and controls in neuropsychology (Panel **a**) and symptoms related to psychopathological (Panel **b**) functioning. The algorithm gives an overall index of the importance of each variable with their respective standard errors and a dichotomic evaluation of “important “ (green boxes) or “not important “ (red boxes). The solid black line represents the mean, the box edges are the first and third quartiles, and the circles are outliers, defined as outside 1.5 times the interquartile range (whiskers) above the upper quartile and below the lower quartile.

After an initial screening and comparison between CHD patients and controls, we used a machine-learning algorithm (Boruta) to find the most influential features distinguishing CHD patients from controls. The algorithm gave an overall index of the importance of each variable with its respective standard errors and a dichotomic evaluation of “important “ or “not important.“ Results are depicted in Fig. 1 for both neuropsychology and psychopathology. We found that the variables most effective in differentiation were (in order of importance) semantic fluency, auditory attention, and visual memory for neuropsychology (Fig. 1a) and thought problems, anxiety/depression, aggressive behavior, and attention problems for symptoms of psychopathology (Fig. 1b). Other important variables are shown (ordered) in Fig. 1. The full variable table is reported in Supplementary Materials’ Tables S1 and S2.

### Cluster analysis

#### Neuropsychology (NPS)

**Figure 2:**
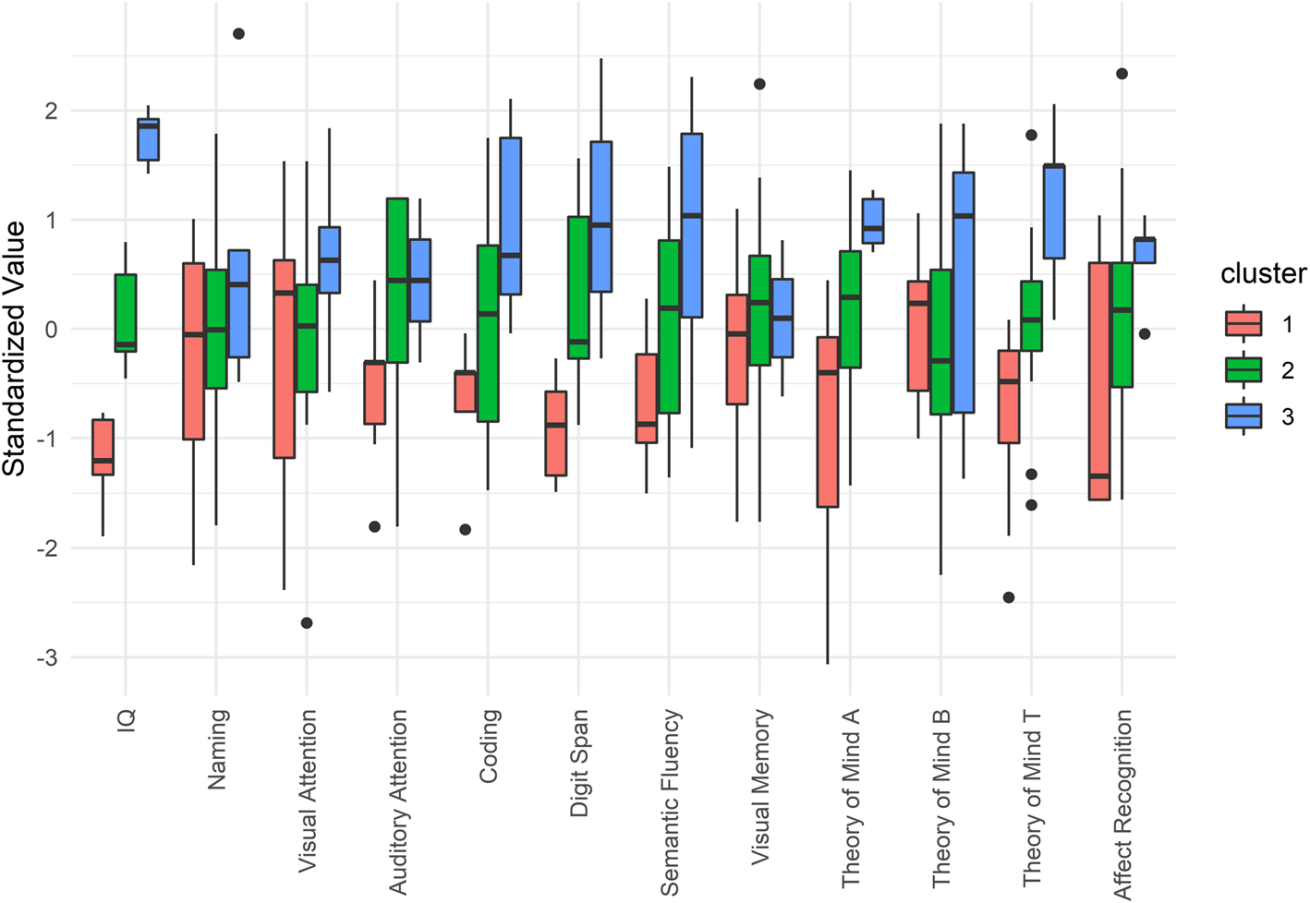
The pattern of neuropsychological functioning obtained through the cluster analysis. The three clusters are represented in three colors (red, green, and blue). The standardized value of each variable is depicted as a boxplot for each cluster. The solid black line represents the mean, the box edges are the first and third quartiles, and the circles are outliers, defined as outside 1.5 times the interquartile range (whiskers) above the upper quartile and below the lower quartile.

Cluster analysis revealed that three clusters explained 55.49% of the point variability of performance at neuropsychological tasks. We evaluated the clusters and determined that the three clusters could be described as follows: The first cluster (Impaired NPS Functioning), accounting for 26% of the sample, exhibited several impairments, particularly in IQ, executive functions, and social skills. The second cluster (Typical NPS Functioning), accounting for 59% of the sample, exhibited average scores in all domains. The third cluster (Good NPS Performance), 15% of the sample, exhibited good performance, particularly in IQ, executive functions, and social skills. The pattern of neuropsychological deficits can be seen in Fig. 2. Numerical data and clinical variable comparisons among clusters are reported in Supplementary Table S2.

#### Symptoms of psychopathology (PSY)

**Figure 3:**
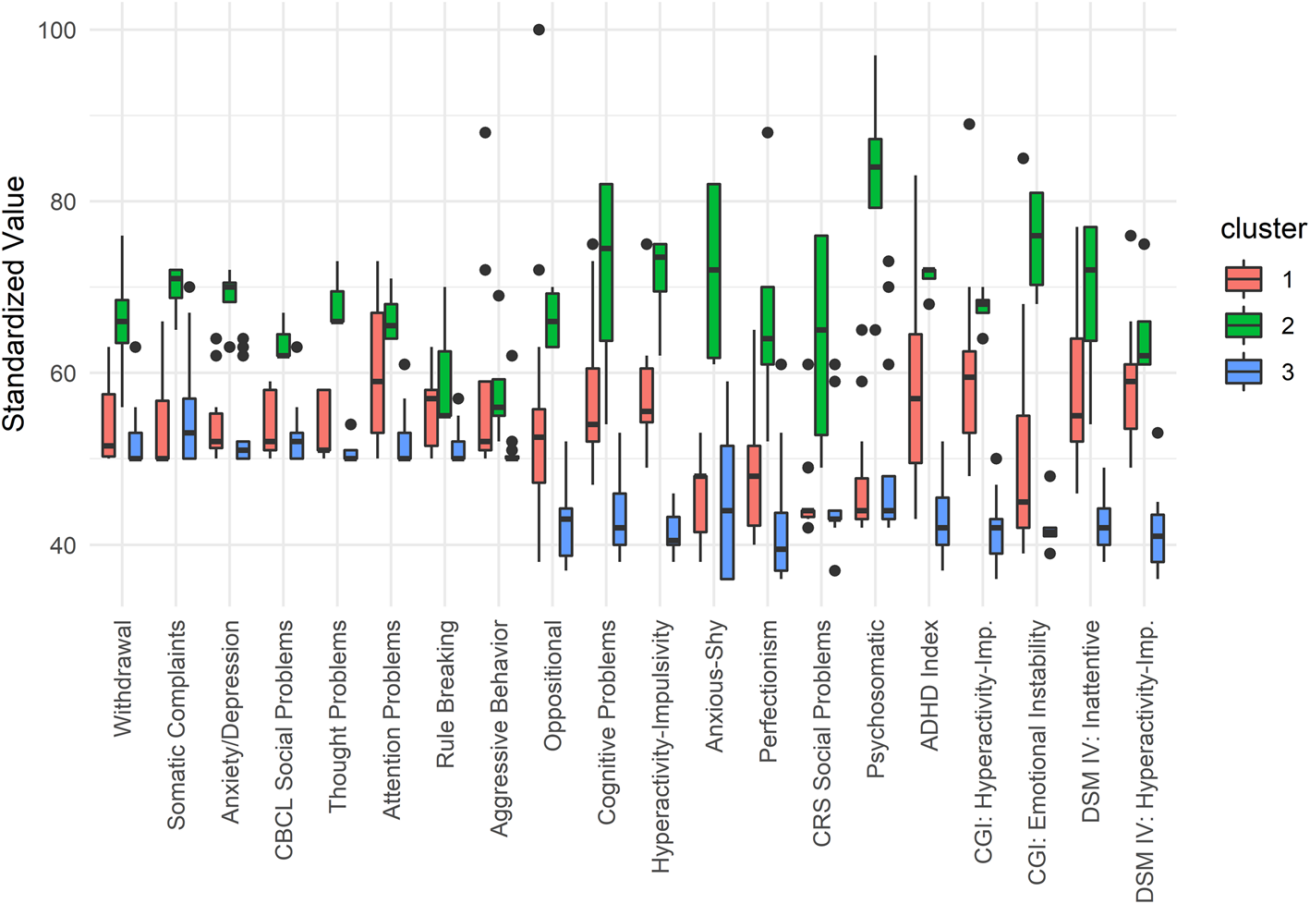
The pattern of psychopathological functioning obtained with the cluster analysis. The three clusters are represented in three colors (red, green, and blue). The standardized value of each variable is depicted as a boxplot for each cluster. The solid black line represents the mean, the box edges are the first and third quartiles, and circles are outliers, defined as outside 1.5 times the interquartile range (whiskers) above the upper quartile and below the lower quartile.

Cluster analysis revealed that three clusters explained 59.02% of the point variability of performance on psychopathological questionnaires. We evaluated the clusters and determined that they could be described as follows: The first cluster (Attention Deficit Hyperactivity Disorder—ADHD), 41% of the sample, exhibited increased inattention, hyperactivity, and impulsivity scores. The second cluster (Global Pathological PSY), 12% of the sample, exhibited clinically relevant scores in most domains. The third cluster (Adequate PSY Functioning), 47% of the sample, exhibited adequate scores in all domains. The pattern of psychopathological scores can be seen in Fig. 3.

The combination of the cluster analyses of neuropsychological and psychopathological scores shows that 31% of patients had no problems and belonged to the clusters designated Typical NPS (23%) or Good NPS Performance (8%) and Adequate PSY Functioning; however, 26% of patients belonged to Typical NPS Functioning but had ADHD, and 8% belonged to Global Pathological PSY; 5% belonged to Good NPS Performance but had ADHD. Finally, 14% exhibited Impaired NPS Functioning alone, 8% with additional ADHD, and 2% with additional Global Pathological PSY. Numerical data and clinical variable comparisons among clusters are reported in Supplementary Table S2.

### Correlation with medical parameters

No significant correlations were found between neuropsychological and psychopathological tasks with clinical parameters in CHD children. No differences in clinical parameters were found between the clusters (Tables S2 and S3).

## Discussion

Our study highlights that CHD survivors, even in the absence of severe disabilities, are at high risk of developing a broad range of neuropsychological and psychopathological dysfunctions. Our purpose was to look for clinically pathological conditions as well as subclinical vulnerabilities, which may emerge from comparison with healthy controls. For a clearer look at the importance of each variable in determining the CHD profile, we implemented a robust machine-learning algorithm to classify variables based on their ability to distinguish CHD patients from controls. We found that the variables presenting the greatest differences were (in order of importance) semantic fluency, auditory attention, and visual memory for neuropsychology and thought problems, anxiety/depression, aggressive behavior, and attention problems for the symptoms of psychopathology. To understand trends related to neuropsychological and psychopathological functioning in the CHD group, we looked for specific homogeneous subtypes using unsupervised, machine learning-based cluster analysis. Interestingly, the neuropsychological tests that differentiated between the groups (CHD *vs* controls) more effectively were those that did *not* differentiate between the clusters. The dysfunctions in these neuropsychological abilities probably represent a common core, while the trend of the three clusters highlights additional variability.

Finally, neither the mean scores nor the clusters appeared influenced or differentiated by the clinical parameters that we selected to account for the perioperative period (pre-, intra-, and post-surgery). One of the strengths of our study is that because a small sample size could be a source of biased analyses, we defined the analysis pipeline a priori and followed it thoroughly. We used a robust, adjusted nonparametric univariate comparison as a first screening of the data to show differences between the CHD patients and controls. The second step was to define which variables were more important to differentiate the groups. To accomplish this task, we used a random forest approach, an ensemble machine-learning method in which multiple independent decision trees are combined to get better predictions. Trees are constructed by randomizing data and variables to obtain the lowest possible correlation among them. The strength of a random forest approach is that it is insensitive to initial correlations among variables (a common problem in every set of psychological evaluation items). Finally, to determine CHD profile clusters, we used a robust clustering approach based on medoids (partitioning around medoids, PAM), in which every cluster is defined after the selection of a representative case. This approach is much less sensitive to influential cases than traditional partitioning methods, such as k-means clustering.

Besides clinically significant impairments, the group comparisons (CHD patients *vs* controls) highlighted significant differences in several domains: intelligence, language, executive skills, and memory. This intrinsic weakness in the neuropsychological performance of CHD patients was confirmed by variable classification and clustering, which established a subgroup of children with low performance, mostly in intelligence, executive functioning, and social skills. Similarly, comparing the groups’ psychopathological scores showed that CHD patients performed worse in many psychological domains. Despite the significant differences, all mean CHD scores were still within the average range in terms of population norms. One reason may be that a healthy control group of typically developing children, growing up in the same period, may offer a more representative reflection of normal variation. Another reason may be that the group’s comparisons allow us to highlight subclinical vulnerabilities. A similar condition may be a predisposition to some personal weaknesses and remain unchanged or get worse over time, as more complex abilities emerge and the cumulative effects of several risk factors act synergistically.

High rates of psychopathological symptoms have been reported in CHD patients^14^. In our study, we found two clinical subtypes of psychopathological profiles: a small subgroup with high symptoms of psychopathology (i.e., widespread clinical elevations in many areas of psychological functioning) and a wider subgroup of patients with ADHD-like profiles, with hyperactivity and impulsivity symptoms as the most represented. ADHD is a disorder associated with white matter injuries, which are typically reported in CHD neonates^15^. It is interesting to note the differences in studies on children born premature, in whom similar patterns of white matter dysmaturity at birth have been reported (for a review, see^15^). Premature children are at high risk of developing ADHD, but the inattentive ADHD subtype has been shown as typical^16^. It is possible that environmental factors impact CHD patients more strongly; compared to premature children, who experience intensive care mostly at birth, CHD children could experience repeated hospitalizations, medical procedures, and follow-up visits throughout childhood. Thus, parent–child interactions may be challenged by repeated exposure to high-risk medical conditions, inducing chronic stress, and less adaptive coping mechanisms, such as overprotective attitudes.

However, contextual factors may not be the sole cause. Among pediatric populations with chronic diseases, CHD patients display higher frequencies of lifetime psychiatric disorders (i.e., 65% *vs* 56% of childhood cancer survivors)^17^. Therefore, the impact of CHD on cerebral circuitries underlying psychopathological vulnerability might be considerable.

The brain develops rapidly in the third trimester and throughout the early postnatal months. During this period, CHD infants are at risk of exposure to hypoxia, neuroinflammation, stress, and clinical procedures requiring general anesthesia. In those children, early structural and microstructural brain abnormalities are already evident in the neonatal period. The mechanism is not well understood, but chronic hypoxia—an effect of some CHDs—could prompt a maturational arrest of oligodendrocyte progenitors, leading to delayed myelination. This abnormal myelination may disrupt neuronal network development via several mechanisms (for a review, see^15^. Subsequently, cardiac surgery may introduce additional risk factors. Such early perturbations of the development of neuronal networks, if sustained, may be responsible for the persistent neurocognitive impairment reported in survivors of CHD. Some late-emerging circuits, particularly in the frontal-subcortical area, have a crucial role in high cognitive functions and psychological abilities^18^, as well as neurologic conditions with psychiatric manifestations (for example^19^).

The heterogeneity of environmental circumstances and differences in the timing of stressful events may explain the broad spectrum of behavioral and psychological symptoms observed in this study’s participants. It has been estimated that known risk factors explain only about 30% of the observed variation in neurodevelopmental outcomes after cardiac surgery in infancy^3^. Interestingly, we did not find medical determinants for our outcomes. To our knowledge, only a few studies focused on specific CHD subgroups have reported associations between neuropsychology/psychopathology and medical or perioperative variables^3,12,13^. It is interesting that the role of medical factors has been so poorly explored in neuropsychological and psychopathological research; there may be an underrepresentation due to the trend to report only positive findings.

In fact, while it is relatively simple to find an association between some medical variables and dichotomous outcome scores, as also reported by our previous works^7,11^, it is challenging to find it related to specific neuropsychological or psychological functions. The developmental trajectories of high-order functions depend on many unpredictable variables, which are strictly interconnected with each other. Individual characteristics, such as genetics, temperament, and specific vulnerability to some impairments or problems, interact with parental variables and illness, hospitalization, and medical procedures. The further we move away from basic cognitive functions, the harder it becomes to find a unique, organic counterpart.

Furthermore, cognition and personality take several years to develop; cumulating effects may take several years to become evident. For this reason, outcomes are highly variable, depending on various factors and timing—there may be a point at which intervention procedures are no longer effective. However, it is not currently understood why some children manifest some symptoms but not others, and how or whether this relates to the developmental time course. Developmental pathways may assume peculiar trajectories, resulting in the high interindividual variability characterizing patients with CHD and other neurological diseases. In our study, interindividual variability manifests itself in highly variable scores in neuropsychological tasks or psychopathological questionnaires, a trend illustrated by the clusters. High interindividual variability may be seen as a methodological limitation, accounting for the differences in the results of many studies, or as a result of the illness itself. Individuals are remarkably diverse, exhibiting variation across a host of behaviors and phenotypes—this is true in typical development, but even more so in atypical development. Furthermore, it confirms the importance of researching early prognostic indicators, as in other pathological conditions^20^.

Our findings should be interpreted in light of potential limiting factors. First, the sample size is small, and even with all the precautions we enacted, this could limit the generalization of our results. Moreover, there is an imbalance in sex between the groups. Therefore, our data should be considered preliminary, and future research should confirm our results.

Regarding the assessment, we did not investigate parental mental states, known to potentially bias assessments of children’s health. Further, an in-depth investigation of psychopathological profiles (based in the present work only on parent ratings) and executive functions could have determined a major understanding of our results.

Finally, our sample covers a broad range of ages. At these ages, executive functioning develops suddenly, new skills emerge, and consequently, the evaluation tasks change substantially for tasks measuring the same executive subcomponents. For this reason, we chose to test “basic “ executive functions, which are relatively mature at an early age.

In conclusion, results on neuropsychological and psychological aspects suggest that a complex framework of cerebral dysfunctions affects children with CHD. Our data are preliminary and should be confirmed by further research with larger samples. The issue deserves attention because childhood neuropsychological and psychological problems may not be present as focused disorders, and therefore, they might be under-recognized. Patients at risk are often identified late because neuropsychological deficits have remained unidentified or unspecified after leading to academic problems. Furthermore, cognitive aspects and medical-clinical characteristics may not fit together in the same puzzle. Causative mechanisms for adverse neurocognitive outcomes are multifactorial, interrelated, cumulative, and likely synergistic over time. Brain maturation, including the refinement of brain networks and myelination, continues through childhood, providing a significant window for recovery and highlighting the need for a lifespan approach to optimizing the outcome trajectory for patients with CHDs. Detailed clinical evaluations focusing on neuropsychological and psychological aspects are a promising path to new neurological and neurobiological research.

## Methods

### Participants

This was a prospective, observational, single-center study of children with CHD. The study was approved by the Institutional Review Board and Ethics Committee, Padova University Hospital, and performed in accordance with relevant regulations. Written informed consent for participation and publication was obtained. Inclusion criteria were: children with complex CHD requiring surgical repair with cardiopulmonary bypass (> 50 min) and hypothermia; elective cardiac surgery, spontaneous breathing, and stable hemodynamic conditions (constant inotropic support if needed, no volume load at admission or during the presurgical hospital stay) before surgery; good Italian linguistic skills; at least 4 years of age; and 18 months from surgery to allow a full recovery. Exclusion criteria included age at surgery > 5 years, liver disease (defined as coagulation factor V < 20%), kidney failure (creatinine clearance < 30%), or known chromosomal abnormalities.

We collected demographic and clinical outcome data prospectively (Table 1). Surgical procedures for CHD repair were classified according to The Society of Thoracic Surgeons-European Association for Cardio-Thoracic Surgery (STAT) scores^21^.

The patients’ clinical characteristics are reported in Table 1.

The controls were recruited in a primary school. The project was presented and asked which families wanted to join freely.

### Outcome assessment

We measured general intelligence using the Wechsler Preschool and Primary Scale of Intelligence III (WPPSI-III^22^) test or the Wechsler Intelligence Scale for Children IV (WISC-IV^23^).

We used the naming test for language^24^; for attention, the visual and auditory attention tests of the NEPSY-II^25^; for memory, the design memory test of the NEPSY-II^25^, which evaluates short-term visuospatial memory; to evaluate executive functions, the coding test of the WISC-IV or WPPSI-III^22,23^, the semantic verbal fluency test, which evaluates the ability to access the lexicon through a categorical cue^24^, and the digit span test of the WISC-IV or WPPSI-III^22,23^, which evaluates working memory; and for social skills, the theory of mind A and B and affect recognition tests of the NEPSY-II^25^.

Detailed descriptions of the tests and procedures are reported in the supplemental material (Table S5).

Parents compiled the following psychopathological questionnaires:

- *Child behavior checklist (CBCL)*^26^ The CBCL is a multiaxial, empirically based set of measures assessing a child ‘s emotional, behavioral, and social problems over the past six months.
- *Conners’ rating scales-revised (CRS-R)*^27^ CPRS-R:L reports parent ratings of children’s behaviors involving problems in seven psychopathological areas: oppositional, inattention, hyperactive, anxious–shy, perfectionism, social problems, and psychosomatic.

### Statistical analysis

Data were expressed as mean (SD), median (Q1-Q3), or percentages. We used a nonparametric Kruskal–Wallis test for patient-control comparisons of continuous variables, and Fisher’s exact test for categorical variables. We used an Armitage trend test for ordered categorical variables. Feature selection relied on a machine-learning algorithm based on random forest (Boruta); the Boruta algorithm aims to identify the relevant predictors that impact the outcome of interest (in our case, belonging to the CHD or control group). It implements a random forest on an augmented set of covariates. Additional covariates, called shadow variables, are copies of the original ones obtained by permuting the observations and thus removing the eventual association with the outcome. For each explanatory variable, an importance measure is computed (i.e., the Z-score), which is the average improvement in the predictive performance of the random forest with the considered explanatory variable divided by its standard deviation. The obtained *important predictors* are those that show Z-scores higher than the one observed for the variable with the maximum Z-score among the shadow variables. The procedure is repeated until an importance measure is assigned to each predictor or the maximum number of random forests is reached. We used the {Boruta} R package^28^ for the analysis. Moreover, to identify underlying homogeneous clusters of CHD patients, we used a robust unsupervised clustering algorithm (PAM) using the {cluster} R package. Included variables of neuropsychology were standardized (Z-scores) and centered before clustering because they were on different scales. The best number of clusters was determined to compare the results of 30 indices with the {NbClus} R package^29^. Variables included for clustering were all the neuropsychology and symptoms of psychopathology parameters except for the “total “ variables to avoid overinflation of single-test sections. We evaluated clinical parameters’ simple linear correlations with neuropsychology and symptoms of psychopathology parameters according to Spearman and corrected multiple comparisons for false discovery rates using the Benjamini–Hochberg method. We used R software v. 4.0 for the analysis and graphics.

## Supplementary Information

Supplementary Information 1.

Supplementary Information 2.

## Data Availability

The datasets generated during and/or analyzed during the current study are available from the corresponding author on reasonable request.
