# Supplementary material for: Detecting neurodevelopmental trajectories in congenital heart diseases with a machine-learning approach
Source: Sci Rep. 2021 Jan 28;11:2574. doi: 10.1038/s41598-021-82328-8 (PMC7843636; doi:10.1038/s41598-021-82328-8)
Supplement: Supplementary file 1 — Supplementary Information 1. [file 41598_2021_82328_MOESM1_ESM.docx]

**Detecting Neurodevelopmental Trajectories in Congenital Heart Diseases with a Machine-Learning Approach**

Elisa Cainelli^1*^, Patrizia S Bisiacchi^1,2*^, Paola Cogo^3^, Massimo Padalino^4^, Manuela Simonato ^5^, Michela Vergine^3^, Corrado Lanera ^6^, Luca Vedovelli^6^

^1^ *Department of General Psychology, University of Padova, Padova, Italy.*

*^2^ Padova Neuroscience Centre, PNC, Padova, Italy*

^3^ *Clinica Pediatrica, Department of Medicine and University Hospital S Maria della Misericordia, University of Udine, Udine, Italy.*

^4^ *Pediatric and Congenital Cardiovascular Surgery Unit, Department of Cardiac, Thoracic and Vascular Sciences, Padova University Hospital, Padova, Italy.*

*^5^ PCare Laboratory, Fondazione Istituto di Ricerca Pediatrica “Citta della Speranza”, Padova, Italy*

*^6^ Unit of Biostatistics, Epidemiology, and Public Health, Department of Cardiac, Thoracic, Vascular Sciences, and Public Health, University of Padova, Padova, Italy.*

Table S5. Description, procedure, and references for all neuropsychological tasks used are reported.

| **Domain** | **Task** | **Description**  **4/24(16%)**  **0/22(0%)**  **1/25(4%)**  **1/24(4%)**  **0/24(0%)**  **3/20(15%)** | **Procedure**  **6/22(27%)**  **2/22(9%)**  **2/21(9%)**  **2/22(9%)**  **4/18(22%)**  **3/21(14%)** | **Reference** |
| --- | --- | --- | --- | --- |
| **Intelligence** | **WISC-IV**  **WPPSI-III** | The Wechsler Intelligence Scales are a group of tests individually administered with standardized procedures used to assess intellectual ability across the lifespan. The test comprises ten primary subtests, organized in ascending difficulty, necessary to obtain a total score (Total IQ). | The test comprises various subtests exploring different cognitive domains (verbal comprehension, fluid and crystalized reasoning, visual-spatial abilities, working memory, processing speed, etc.). Results are provided in quotients (100 mean, 15 standard deviation). | Wechsler, 2002  Wechsler, 2003 |
| **Language** | **Naming** | Naming is a test evaluating the ability to retrieve the name of pictures promptly. The test is composed of 20 (6-11 years) or 88 (12-16 years) items. The performance is measured in the total number of words. | The patient is shown target stimuli and asked to identify each target item within a 20-second interval per trial. Results are provided in z scores (0 mean, 1 standard deviation). | Bisiacchi, Cendron, Gugliotta, Tressoldi, & Vio, 2005) |
| **Memory** | **Design Memory** | This subtest is designed to assess spatial memory for novel visual material. | The child is shown a grid with four to ten  designs on a page, which is then removed from view. The child selects the designs from a set of cards and places the  cards on a grid in the same location as previously shown. Results are provided in scaled score (10 mean, 3 standard deviation). | Korkman et al., 2007 |
| **Attention** | **Visual attention** | Visual Attention is designed to assess selective visual attention and the ability to sustain it (vigilance). | This is a time-limited (3 minutes) task. The child has to search two target faces between several distractors' faces. Results are provided in scaled score (10 mean, 3 standard deviation). | Korkman et al., 2007 |
|  | **Auditory Attention** | Auditory Attention is designed to assess selective auditory attention and the ability to sustain it (vigilance). | The child listens to a series of words and touches the appropriate circle when he or she hears a target word. Results are provided in percentiles (pathological < 5°). For this work, percentiles are transformed into an ordinale scale, in which 1= 1-5°, 2=6-10°, 3=11-25°,4= 26-50°, 5=51-75°, 6>75°. | Korkman et al., 2007 |
| **Executive Functions** | **Semantic fluency** | Semantic fluency evaluates the retrieval of words from conceptual (semantic) memory. This word retrieval process requires the operation of organized neural networks to access and decode long-term memory stores. The used categories are colors, animals, fruits, and city, and the performance is measured in the total number of words. | The experimenter asks the child to say as many names as possible of items belonging to each category in a 1-minute trial. Results are provided in z scores (0 mean, 1 standard deviation). | Bisiacchi, Cendron, Gugliotta, Tressoldi, & Vio, 2005) |
|  | **Coding** | Coding measures visual-motor dexterity, associative nonverbal learning, and nonverbal short-term memory. Fine-motor dexterity, speed, accuracy and ability to manipulate a pencil contribute to task success; perceptual organization is also important. | Coding is a timed subtest. Children are given a worksheet with a top line containing the key (symbols associated with figures for children < 8 years and symbols associated with numbers for children from 8 years). They must complete with the correct symbol all the next lines. Results are provided in scaled score (10 mean, 3 standard deviation). | Wechsler, 2002, 2003 |
|  | **Digit span** | A digit-span task is used to measure working memory's number storage capacity. Digit span forward is mainly a measure of [short-term memory](https://en.wikipedia.org/wiki/Short-term_memory). The participant's span is the longest number of sequential digits that can accurately be remembered. The sequence started from 3 numbers. Backward digit span is a more challenging variation that involves recalling items in reverse order. It is a measure of working memory as well as short-term memory. The sequence started from 2 numbers. | Participants hear a sequence of numerical digits and are tasked to recall the sequence correctly, with increasingly longer sequences being tested in each trial. In the backward version, the participant is asked to recall the sequence in reverse order. Results are provided in scaled score (10 mean, 3 standard deviation). | Wechsler, 2002, 2003 |
| **Social skills** | **Affect Recognition** | This subtest is designed to assess the ability to recognize affect (happy, sad, anger, fear, disgust, and neutral) from photographs of children’s faces in four different tasks. | In one task, the child simply states whether or not two photographs depict faces with the same affect. In a second task, he or she selects two photographs of faces with the same affect from 3–4 photographs. In a third task, the child selects one of the four faces that depicts the same affect as a face at the top of the page. Finally, the child is briefly shown a face and, from memory, selects two photographs that depict the same affect as the face previously shown. Results are provided in scaled score (10 mean, 3 standard deviation). | Korkman et al., 2007 |
|  | **Theory of Mind** | This subtest is designed to assess the ability to understand mental functions such as belief, intention, deception, emotion, imagination, and pretending, as well as the ability to understand that others have their own thoughts, ideas, and feelings that may be different from one’s own and the ability to understand how emotion relates to social context and to recognize the appropriate affect given various social contexts. | In the Verbal task, the child is read various scenarios or shown pictures and is then asked questions that require knowledge of another individual’s point of view to answer correctly. In the Contextual task, the child is shown a picture depicting a social context and asked to select a photograph from four options that depict the appropriate affect of one of the people in the picture. Results are provided in scaled score (10 mean, 3 standard deviation). | Korkman et al., 2007 |
