# Supplementary material for: Detecting neurodevelopmental trajectories in congenital heart diseases with a machine-learning approach
Source: Sci Rep. 2021 Jan 28;11:2574. doi: 10.1038/s41598-021-82328-8 (PMC7843636; doi:10.1038/s41598-021-82328-8)
Supplement: Supplementary file 2 — Supplementary Information 2. [file 41598_2021_82328_MOESM2_ESM.docx]

**Detecting Neurodevelopmental Trajectories in Congenital Heart Diseases with a Machine-Learning Approach**

Elisa Cainelli^1*^, Patrizia S Bisiacchi^1,2*^, Paola Cogo^3^, Massimo Padalino^4^, Manuela Simonato ^5^, Michela Vergine^3^, Corrado Lanera ^6^, Luca Vedovelli^6^

^1^ *Department of General Psychology, University of Padova, Padova, Italy.*

*^2^ Padova Neuroscience Centre, PNC, Padova, Italy*

^3^ *Clinica Pediatrica, Department of Medicine and University Hospital S Maria della Misericordia, University of Udine, Udine, Italy.*

^4^ *Pediatric and Congenital Cardiovascular Surgery Unit, Department of Cardiac, Thoracic and Vascular Sciences, Padova University Hospital, Padova, Italy.*

*^5^ PCare Laboratory, Fondazione Istituto di Ricerca Pediatrica “Citta della Speranza”, Padova, Italy*

*^6^ Unit of Biostatistics, Epidemiology, and Public Health, Department of Cardiac, Thoracic, Vascular Sciences, and Public Health, University of Padova, Padova, Italy.*

**Table S1**

Boruta full numeric results for neuropsychology variables. Imp: importance, decision: if the variable is important to differentiate the CHD and controls groups.

**
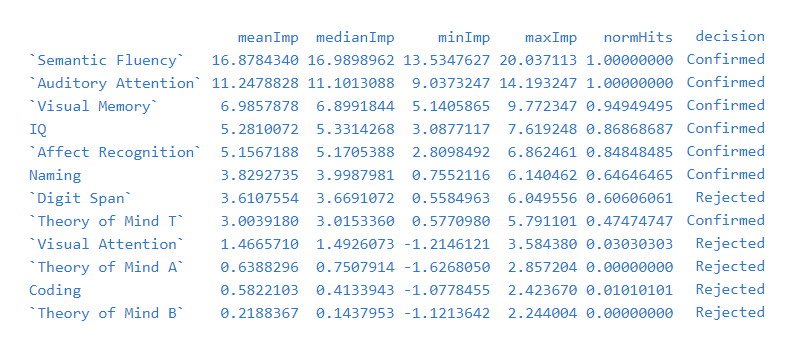
**

**Table S2**

Boruta full numeric results for psychopatology variables. Imp: importance, decision: if the variable is important to differentiate the CHD and controls groups.


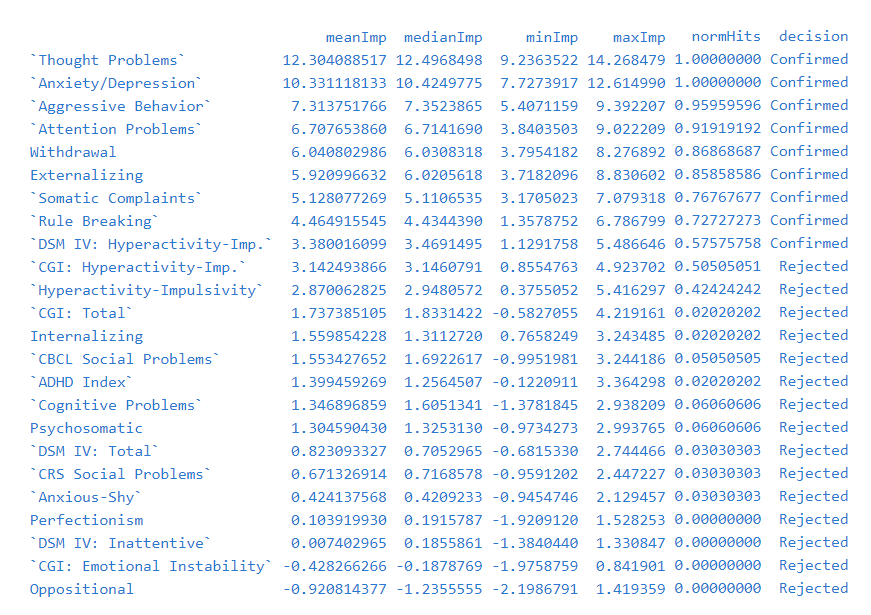


**Table S3**.

*Neuropsychology and clinical variables comparisons after clustering.*

Clinical variables were not included in the clustering but evaluated *a posteriori*. Results are presented in Z-scores for neuropsychology variables and in the original scales for clinical variables. P-values are adjusted for false discovery rate.

| **Cluster** | 1 (N=9) | 2 (N=20) | 3 (N=5) | p value |
| --- | --- | --- | --- | --- |
| **IQ** |  |  |  | **< 0.001** |
| - Median | -1.2 | -0.1 | 1.9 |  |
| - Q1,Q3 | -1.3, -0.8 | -0.2, 0.5 | 1.5, 1.9 |  |
| - Mean | -1.2 (0.4) | 0.1 (0.4) | 1.8 (0.3) |  |
| **Coding** |  |  |  | 0.064 |
| - Median | -0.4 | 0.1 | 0.7 |  |
| - Q1,Q3 | -0.8, -0.4 | -0.8, 0.8 | 0.3, 1.7 |  |
| - Mean | -0.6 (0.5) | 0.0 (1.0) | 1.0 (0.9) |  |
| **Digit Span** |  |  |  | **0.021** |
| - Median | -0.9 | -0.1 | 1.0 |  |
| - Q1,Q3 | -1.3, -0.6 | -0.3, 1.0 | 0.3, 1.7 |  |
| - Mean | -0.9 (0.5) | 0.2 (0.8) | 1.1 (1.4) |  |
| **Naming** |  |  |  | 0.718 |
| - Median | -0.1 | -0.0 | 0.4 |  |
| - Q1,Q3 | -1.0, 0.6 | -0.5, 0.5 | -0.3, 0.7 |  |
| - Mean | -0.3 (1.1) | -0.0 (0.9) | 0.6 (1.3) |  |
| **Semantic Fluency** |  |  |  | 0.109 |
| - Median | -0.9 | 0.2 | 1.0 |  |
| - Q1,Q3 | -1.0, -0.2 | -0.8, 0.8 | 0.1, 1.8 |  |
| - Mean | -0.7 (0.6) | 0.1 (0.9) | 0.8 (1.4) |  |
| **Visual Attention** |  |  |  | 0.671 |
| - Median | 0.3 | 0.0 | 0.6 |  |
| - Q1,Q3 | -1.2, 0.6 | -0.6, 0.4 | 0.3, 0.9 |  |
| - Mean | -0.2 (1.3) | -0.1 (0.9) | 0.6 (0.9) |  |
| **Theory of Mind A** |  |  |  | **0.015** |
| - Median | -0.4 | 0.3 | 0.9 |  |
| - Q1,Q3 | -1.6, -0.1 | -0.4, 0.7 | 0.8, 1.2 |  |
| - Mean | -0.8 (1.2) | 0.1 (0.7) | 1.0 (0.2) |  |
| **Theory of Mind B** |  |  |  | 0.829 |
| - Median | 0.2 | -0.3 | 1.0 |  |
| - Q1,Q3 | -0.6, 0.4 | -0.8, 0.5 | -0.8, 1.4 |  |
| - Mean | -0.0 (0.8) | -0.1 (1.0) | 0.4 (1.4) |  |
| **Theory of Mind T** |  |  |  | **0.017** |
| - Median | -0.5 | 0.1 | 1.5 |  |
| - Q1,Q3 | -1.0, -0.2 | -0.2, 0.4 | 0.6, 1.5 |  |
| - Mean | -0.7 (0.9) | 0.0 (0.8) | 1.2 (0.8) |  |
| **Affect Recognition** |  |  |  | 0.155 |
| - Median | -1.3 | 0.2 | 0.8 |  |
| - Q1,Q3 | -1.6, 0.6 | -0.5, 0.6 | 0.6, 0.8 |  |
| - Mean | -0.6 (1.1) | 0.1 (0.9) | 0.6 (0.4) |  |
| **Auditory Attention** |  |  |  | 0.492 |
| - Median | -0.3 | 0.4 | 0.4 |  |
| - Q1,Q3 | -0.9, -0.3 | -0.3, 1.2 | 0.1, 0.8 |  |
| - Mean | -0.6 (0.8) | 0.2 (1.1) | 0.4 (0.8) |  |
| **Visual Memory** |  |  |  | 0.854 |
| - Median | -0.0 | 0.2 | 0.1 |  |
| - Q1,Q3 | -0.7, 0.3 | -0.3, 0.7 | -0.3, 0.5 |  |
| - Mean | -0.2 (0.9) | 0.1 (1.1) | 0.1 (1.0) |  |
| **Clinical Variables** |  |  |  |  |
| **STAT category** |  |  |  | 0.788 |
| - 1 | 2 (22.2%) | 1 (5.0%) | 2 (40.0%) |  |
| - 2 | 2 (22.2%) | 9 (45.0%) | 2 (40.0%) |  |
| - 3 | 2 (22.2%) | 4 (20.0%) | 0 (0.0%) |  |
| - 4 | 2 (22.2%) | 4 (20.0%) | 1 (20.0%) |  |
| - 5 | 1 (11.1%) | 2 (10.0%) | 0 (0.0%) |  |
| **Age at sugery (m)** |  |  |  | 0.718 |
| - Median | 6.9 | 3.0 | 25.3 |  |
| - Q1,Q3 | 4.5, 11.2 | 0.3, 21.6 | 6.2, 44.0 |  |
| - Mean | 16.1 (22.1) | 14.1 (20.5) | 25.1 (22.1) |  |
| **Pre-surgery**  **Oxy. Sat. (%)** |  |  |  | 0.785 |
| - Median | 88.0 | 90.0 | 98.0 |  |
| - Q1,Q3 | 84.0, 96.0 | 85.8, 97.2 | 87.5, 99.0 |  |
| - Mean | 88.9 (9.2) | 90.9 (6.7) | 93.7 (7.4) |  |
| **Surgery**  **Temp. Nadir (°C)** |  |  |  | 0.677 |
| - Median | 31.0 | 31.2 | 34.0 |  |
| - Q1,Q3 | 27.0, 32.7 | 28.0, 32.2 | 32.0, 34.0 |  |
| - Mean | 29.1 (5.3) | 30.1 (3.4) | 31.7 (4.1) |  |
| **Cardiopulmonary**  **Bypass time (min)** |  |  |  | 0.718 |
| - Median | 143.0 | 125.0 | 84.0 |  |
| - Q1,Q3 | 90.0, 158.0 | 100.5, 144.5 | 57.0, 130.0 |  |
| - Mean | 132.9 (62.7) | 128.4 (45.5) | 99.2 (52.7) |  |
| **Time at cerebral**  **Sat. <45%, NIRS (min)** |  |  |  | 0.829 |
| - Median | 2.2 | 12.9 | 5.0 |  |
| - Q1,Q3 | 0.0, 25.0 | 0.0, 19.7 | 0.0, 7.1 |  |
| - Mean | 18.7 (27.0) | 22.0 (31.7) | 6.5 (8.4) |  |
| **Mean surgery lactates**  **(mmol/L)** |  |  |  | 0.854 |
| - Median | 1.7 | 2.7 | 2.0 |  |
| - Q1,Q3 | 1.5, 2.9 | 1.4, 3.7 | 1.4, 2.4 |  |
| - Mean | 2.3 (1.3) | 2.9 (1.8) | 2.3 (1.3) |  |
| **Days in intensive care** |  |  |  | 0.718 |
| - Median | 3.0 | 5.0 | 2.0 |  |
| - Q1,Q3 | 2.0, 7.0 | 2.5, 5.0 | 1.0, 5.0 |  |
| - Mean | 11.1 (19.5) | 5.7 (6.0) | 2.8 (2.0) |  |

**Table S4**.

*Psychopathology and clinical variables comparisons after clustering.*

Clinical variables were not included in the clustering but evaluated *a posteriori*. Results are presented in Z-scores for psychopathology variables and in the original scales for clinical variables. P-values are adjusted for false discovery rate.

| **Cluster** | 1 (N=14) | 2 (N=4) | 3 (N=16) | p value |  |
| --- | --- | --- | --- | --- | --- |
| **Oppositional** |  |  |  | **< 0.001** | |
| Median | 52.5 | 66.0 | 43.0 |  | |
| Q1,Q3 | 47.2, 55.8 | 63.0, 69.2 | 38.8, 44.2 |  | |
| Mean | 56.0 (15.0) | 66.2 (3.8) | 42.8 (4.7) |  | |
| **Cognitive Problems** |  |  |  | **< 0.001** | |
| Median | 54.0 | 74.5 | 42.0 |  | |
| Q1,Q3 | 52.0, 60.5 | 63.8, 82.0 | 40.0, 46.0 |  | |
| Mean | 57.2 (9.2) | 71.2 (13.5) | 42.9 (4.1) |  | |
| **Hyperactivity-Impulsivity** |  |  |  | **< 0.001** | |
| Median | 55.5 | 73.5 | 40.5 |  | |
| Q1,Q3 | 54.2, 60.5 | 69.5, 75.0 | 40.0, 43.2 |  | |
| Mean | 56.9 (6.7) | 71.0 (6.2) | 41.4 (2.3) |  | |
| **Anxious-Shy** |  |  |  | **0.009** | |
| Median | 48.0 | 72.0 | 44.0 |  | |
| Q1,Q3 | 41.5, 48.0 | 61.8, 82.0 | 36.0, 51.5 |  | |
| Mean | 45.6 (4.9) | 71.8 (11.8) | 44.3 (7.9) |  | |
| **Perfectionism** |  |  |  | **0.002** | |
| Median | 48.0 | 64.0 | 39.5 |  | |
| Q1,Q3 | 42.2, 51.5 | 61.0, 70.0 | 37.0, 43.8 |  | |
| Mean | 48.9 (7.7) | 67.0 (15.1) | 42.1 (7.0) |  | |
| **CRS Social Problems** |  |  |  | **0.015** | |
| Median | 44.0 | 65.0 | 43.0 |  |  |
| Q1,Q3 | 43.2, 44.0 | 52.8, 76.0 | 43.0, 44.0 |  |  |
| Mean | 45.4 (5.0) | 63.8 (14.3) | 44.6 (6.4) |  |  |
| **Psychosomatic** |  |  |  | **0.012** | |
| Median | 44.0 | 84.0 | 44.0 |  | |
| Q1,Q3 | 43.0, 47.8 | 79.2, 87.2 | 43.0, 48.0 |  | |
| Mean | 47.0 (7.0) | 82.5 (13.2) | 48.9 (9.9) |  | |
| **ADHD Index** |  |  |  | **< 0.001** | |
| Median | 57.0 | 72.0 | 42.0 |  | |
| Q1,Q3 | 49.5, 64.5 | 71.0, 72.0 | 40.0, 45.5 |  | |
| Mean | 58.3 (11.3) | 71.0 (2.0) | 43.1 (4.1) |  | |
| **CGI: Hyperactivity-Imp.** |  |  |  | **< 0.001** | |
| Median | 59.5 | 68.0 | 42.0 |  | |
| Q1,Q3 | 53.0, 62.5 | 67.0, 68.5 | 39.0, 43.0 |  | |
| Mean | 59.9 (10.7) | 67.5 (2.5) | 41.9 (3.5) |  | |
| **CGI: Emotional Instability** |  |  |  | **0.002** | |
| Median | 45.0 | 76.0 | 41.5 |  | |
| Q1,Q3 | 42.0, 55.0 | 70.2, 81.0 | 41.0, 42.0 |  | |
| Mean | 50.4 (12.8) | 75.2 (6.8) | 41.9 (2.6) |  | |
| **DSM IV: Inattentive** |  |  |  | **< 0.001** | |
| Median | 55.0 | 72.0 | 42.0 |  | |
| Q1,Q3 | 52.0, 64.0 | 63.8, 77.0 | 40.0, 44.2 |  | |
| Mean | 58.0 (10.3) | 68.8 (10.9) | 42.7 (3.0) |  | |
| **DSM IV: Hyperactivity-Imp.** |  |  |  | **< 0.001** | |
| Median | 59.0 | 62.0 | 41.0 |  |  |
| Q1,Q3 | 53.5, 61.0 | 61.0, 66.0 | 38.0, 43.5 |  |  |
| Mean | 58.4 (7.6) | 65.0 (6.7) | 41.4 (4.3) |  |  |
| **Withdrawal** |  |  |  | **0.019** |  |
| Median | 51.5 | 66.0 | 50.0 |  |  |
| Q1,Q3 | 50.2, 57.5 | 63.5, 68.5 | 50.0, 53.0 |  |  |
| Mean | 54.0 (4.8) | 66.0 (8.2) | 53.2 (5.1) |  |  |
| **Somatic Complaints** |  |  |  | **0.011** |  |
| Median | 50.0 | 71.0 | 53.0 |  |  |
| Q1,Q3 | 50.0, 56.8 | 68.8, 72.0 | 50.0, 57.0 |  |  |
| Mean | 53.7 (5.6) | 69.8 (3.3) | 54.8 (6.2) |  |  |
| **Anxiety/Depression** |  |  |  | **0.005** |  |
| Median | 52.0 | 70.0 | 51.0 |  |  |
| Q1,Q3 | 51.2, 55.2 | 68.2, 70.5 | 50.0, 52.0 |  |  |
| Mean | 53.9 (4.3) | 68.8 (3.9) | 53.0 (5.0) |  |  |
| **CBCL Social Problems** |  |  |  | **0.040** |  |
| Median | 52.0 | 62.0 | 52.0 |  |  |
| Q1,Q3 | 51.0, 58.0 | 62.0, 64.5 | 50.0, 53.0 |  |  |
| Mean | 54.1 (4.0) | 63.7 (2.9) | 52.5 (3.6) |  |  |
| **Thought Problems** |  |  |  | **0.010** |  |
| Median | 51.0 | 66.0 | 50.0 |  |  |
| Q1,Q3 | 51.0, 58.0 | 66.0, 69.5 | 50.0, 51.0 |  |  |
| Mean | 53.9 (3.9) | 68.3 (4.0) | 51.2 (1.7) |  |  |
| **Attention Problems** |  |  |  | **0.001** |  |
| Median | 59.0 | 65.5 | 50.0 |  |  |
| Q1,Q3 | 53.0, 67.0 | 64.0, 68.0 | 50.0, 53.0 |  |  |
| Mean | 60.4 (8.3) | 66.5 (3.3) | 51.8 (3.1) |  |  |
| **Rule Breaking** |  |  |  | **0.021** |  |
| Median | 57.0 | 55.0 | 50.0 |  |  |
| Q1,Q3 | 51.5, 58.0 | 55.0, 62.5 | 50.0, 52.0 |  |  |
| Mean | 55.6 (4.8) | 60.0 (8.7) | 51.3 (2.3) |  |  |
| **Aggressive Behavior** |  |  |  | **0.002** |  |
| Median | 52.0 | 56.0 | 50.0 |  |  |
| Q1,Q3 | 51.0, 59.0 | 55.0, 59.2 | 50.0, 50.2 |  |  |
| Mean | 57.2 (10.7) | 58.2 (7.4) | 51.0 (3.0) |  |  |
| **STAT category** |  |  |  | 0.274 |  |
| 1 | 3 (21.4%) | 1 (25.0%) | 0 (0.0%) |  |  |
| 2 | 7 (50.0%) | 1 (25.0%) | 6 (37.5%) |  |  |
| 3 | 2 (14.3%) | 0 (0.0%) | 4 (25.0%) |  |  |
| 4 | 2 (14.3%) | 2 (50.0%) | 3 (18.8%) |  |  |
| 5 | 0 (0.0%) | 0 (0.0%) | 3 (18.8%) |  |  |
| **Age at sugery (m)** |  |  |  | 0.614 |  |
| Median | 4.7 | 2.4 | 6.0 |  |  |
| Q1,Q3 | 2.0, 10.6 | 0.2, 6.2 | 0.4, 30.6 |  |  |
| Mean | 12.3 (18.1) | 4.0 (5.2) | 17.5 (22.5) |  |  |
| **Pre-surgery**  **Oxy. Sat. (%)** |  |  |  | 0.485 |  |
| Median | 91.0 | 96.0 | 87.5 |  |  |
| Q1,Q3 | 86.0, 96.8 | 93.1, 97.8 | 84.8, 96.5 |  |  |
| Mean | 90.6 (7.5) | 94.9 (5.3) | 89.0 (8.0) |  |  |
| **Surgery**  **Temp. Nadir (°C)** |  |  |  | 0.553 |  |
| Median | 32.0 | 30.0 | 29.6 |  |  |
| Q1,Q3 | 31.1, 32.7 | 27.1, 32.0 | 27.8, 33.0 |  |  |
| Mean | 30.6 (4.4) | 29.1 (3.6) | 29.6 (3.8) |  |  |
| **Cardiopulmonary**  **Bypass time (min)** |  |  |  | 0.830 |  |
| Median | 125.0 | 143.5 | 136.0 |  |  |
| Q1,Q3 | 102.0, 154.5 | 129.8, 151.8 | 92.2, 149.0 |  |  |
| Mean | 127.4 (54.6) | 138.0 (35.3) | 128.9 (50.7) |  |  |
| **Time at cerebral**  **Sat. <45%, NIRS (min)** |  |  |  | 0.648 |  |
| Median | 13.7 | 5.0 | 7.7 |  |  |
| Q1,Q3 | 0.0, 30.1 | 0.0, 10.7 | 0.0, 21.0 |  |  |
| Mean | 21.6 (27.5) | 5.7 (6.6) | 21.8 (33.5) |  |  |
| **Mean surgery lactates**  **(mmol/L)** |  |  |  | 0.963 |  |
| Median | 2.0 | 2.5 | 2.4 |  |  |
| Q1,Q3 | 1.5, 3.6 | 1.6, 3.5 | 1.4, 4.5 |  |  |
| Mean | 2.3 (1.1) | 2.6 (1.5) | 3.0 (2.0) |  |  |
| **Days in intensive care** |  |  |  | 0.553 |  |
| Median | 4.5 | 4.5 | 5.0 |  |  |
| Q1,Q3 | 2.0, 5.0 | 3.2, 5.0 | 2.5, 7.0 |  |  |
| Mean | 7.7 (15.7) | 3.8 (1.9) | 7.0 (6.9) |  |  |
